# Beta-Alanine and Aquagenic Pruritus: Proposed Neuroimmune Mechanism

**DOI:** 10.2196/90737

**Published:** 2026-03-25

**Authors:** Natalie Piserchio, Bailey Baratta, Benjamin Brooks, Brandon Muse, Katelin Ball

**Affiliations:** 1 Rocky Vista University, 8401 S Chambers Rd, Englewood, CO, 80112, United States, 1 (303) 373-2008; 2 Internal Medicine Residency HEB/Denton, Texas Health Resources, Bedford, TX, United States; 3 Department of Biomedical Sciences, Rocky Vista University, Ivins, UT, United States; 4 Department of Obstetrics and Gynecology, Orlando Health, Orlando, FL, United States; 5 Department of Neurology, University of New Mexico, Albuquerque, NM, United States

**Keywords:** aquagenic pruritus, beta-alanine, primary aquagenic pruritus, MrgprD, nonhistaminergic itch, mast-cell hyperresponsiveness, neuroimmune signaling, glutamatergic signaling, pruriceptive pathways, neurocutaneous mechanisms, itch modulation, MAS-related G protein–coupled receptor D

## Abstract

Aquagenic pruritus (AP) is a rare itch disorder with limited effective treatments, and emerging clinical observations suggest that oral β-alanine may reduce symptoms. The purpose of this viewpoint is to propose a biologically plausible mechanism through which β-alanine may alleviate primary AP. We reviewed published case reports and patient-reported survey data describing β-alanine use in AP and integrated these clinical observations with experimental data on MAS-related G protein–coupled receptor D (MrgprD)–expressing sensory neurons and their role in mast-cell regulation. Published case reports describe marked improvement in water-induced pruritus following prophylactic oral β-alanine administration, and a recent survey of patients with idiopathic AP reported substantial symptom relief among β-alanine users. Preclinical data indicate that MrgprD-neuronal glutamate release suppresses mast cell hyperresponsiveness, suggesting a potential pathway for the observed antipruritic effect. Additional mechanisms, including β-alanine metabolism to carnosine and its potential mast cell–stabilizing effects, may also contribute. β-alanine may act through modulation of a nonhistaminergic neuroimmune circuit and represents a promising therapeutic candidate for further investigation in AP.

Aquagenic pruritus (AP) is a rare skin disorder characterized by intense itching, tingling, or burning after contact with water, which can significantly impact an individual’s quality of life [1]. Although AP is classically associated with polycythemia vera, idiopathic (primary) AP likely represents the most common form in the general population [2]. Therapeutic approaches to alleviate this condition remain challenging and often ineffective. Existing treatments primarily focus on symptom relief through topical emollients, phototherapy, antihistamines, and avoidance of water exposure, but they provide only partial relief and do not address the underlying mechanisms triggering the pruritus [1].

The pathogenesis of idiopathic AP is incompletely understood. Several mechanisms have been proposed, including abnormal mast cell activation, dysregulated cutaneous nerve signaling, and altered neuroimmune communication within the skin [3]. A small biopsy study found increased acetylcholinesterase activity in eccrine-associated nerve fibers after water exposure in patients with AP and polycythemia vera, suggesting increased acetylcholine release and possible involvement of eccrine-associated pathways in AP pathogenesis [4]. AP has also been described in familial cases, suggesting a potential genetic predisposition [1].

Recent case reports have shed light on the role of β-alanine, a nonessential amino acid, in modulating the sensation of itch. β-alanine is a known agonist at the pruriceptive receptor MAS-related G protein–coupled receptor D (MrgprD) and induces a weak nonhistaminergic itch in humans and mice [5]. A survey-based study investigating β-alanine supplementation among 75 patients with idiopathic AP reported that 70.7% of participants who used β-alanine described substantial symptom relief, with an average relief score of 8.84 out of 10 (95% CI 8.52‐9.16) [6].

In one case report, a 33-year-old adult male with primary AP found that prophylactic oral β-alanine taken 5 to 15 minutes before water exposure markedly reduced pruritus with sustained improvement at the 20-week follow-up [5]. In a second case, a 16-year-old male with AP refractory to traditional treatments also found relief from oral β-alanine taken prior to water exposure [7].

In these reports, β-alanine was administered orally in powder form [5,7]. Survey data suggest that patients most commonly use doses averaging approximately 1.59 g per day during acute exacerbations. In this survey, 66% of users preferred powder formulations and 73.6% reported taking β-alanine on an as-needed basis [6].

The mechanism responsible for this improvement is unknown, and because MrgprD activation is typically pruritogenic, the antipruritic effect is unlikely to result from direct receptor agonism. Other mechanisms may also contribute to the observed therapeutic benefit. For example, β-alanine is a precursor to carnosine, a dipeptide with antioxidant and intracellular pH-buffering properties that may help stabilize mast cell responses. These effects could complement or act independently of MrgprD-mediated neural pathways [6].

Although the mechanisms underlying primary AP are not fully established, the delay between water exposure and itch onset reported in many patients may suggest a nonhistaminergic process involving abnormal neuroimmune interactions between sensory afferents and epidermal or immune cells such as keratinocytes, basophils, or mast cells [5]. A mouse study from 2021 demonstrated that MrgprD-expressing cutaneous sensory neurons help maintain skin homeostasis by releasing glutamate, which dampens mast cell hyperresponsiveness [3]. One possibility is that β-alanine may transiently enhance MrgprD-neuronal activity enough to increase local glutamate release, thereby strengthening this homeostatic inhibitory circuit. In primary AP, where mast cell hyperresponsiveness and nonhistaminergic pathways are suspected, enhanced MrgprD-mediated glutamatergic signaling could suppress mast cell activation and reduce downstream pruriceptive input. Recent work from the same research group further supports this model by demonstrating that glutamate can act directly on both mouse and human mast cells to suppress their activation, reinforcing the potential role of glutamatergic signaling in cutaneous immune homeostasis [8].

Available patient-reported data suggest that β-alanine is generally well tolerated, with transient paresthesia being the most frequently reported side effect. This is consistent with the known safety profile of β-alanine in sports nutrition studies and is typically mild and self-limited. Importantly, 90% of surveyed users reported sustained therapeutic benefit without loss of efficacy over time, suggesting minimal tachyphylaxis [6,9].

Although speculative, this mechanism aligns with both the delayed nature of AP symptoms reported by some individuals and the observed clinical benefit of prophylactic β-alanine administration. Given the debilitating nature of AP and the scarcity of effective therapies, the emerging evidence supporting β-alanine as a fast-acting, well-tolerated, inexpensive, and accessible intervention is noteworthy.

We encourage further controlled studies to characterize optimal dosing, duration of effect, long-term safety, and the underlying neurocutaneous mechanisms. If β-alanine consistently alleviates symptoms in primary AP, this observation may also support the concept that non–FcεR-mediated mast cell activation and neuroimmune dysregulation play important roles in disease pathogenesis.

β-alanine may represent a promising therapeutic avenue for a condition that currently lacks reliable treatment options. These observations highlight the need to further explore β-alanine as a targeted modulator of nonhistaminergic itch pathways in primary AP.

## Abbreviations

AP: aquagenic pruritus
MrgprD: MAS-related G protein–coupled receptor D

## References

[1] Siegel FP, Tauscher J, Petrides PE (2013). Aquagenic pruritus in polycythemia vera: characteristics and influence on quality of life in 441 patients. Am J Hematol.

[2] Steinman HK, Greaves MW (1985). Aquagenic pruritus. J Am Acad Dermatol.

[3] Zhang S, Edwards TN, Chaudhri VK (2021). Nonpeptidergic neurons suppress mast cells via glutamate to maintain skin homeostasis. Cell.

[4] Bircher AJ, Meier-Ruge W (1988). Aquagenic pruritus: water-induced activation of acetylcholinesterase. Arch Dermatol.

[5] Alinaghi F, Elberling J (2024). Primary aquagenic pruritus successfully treated by β-alanine: a case report. Case Rep Dermatol.

[6] Concistrè A (2024). Beta-alanine as a potential treatment for aquagenic pruritus: an online social media-based survey study. Acta Dermatovenerol Croat.

[7] Degala J, Degala A (2025). The use of beta-alanine for the management of aquagenic pruritus. Ann Allergy Asthma Immunol.

[8] Zhang YR, Keshari S, Kurihara K (2024). Agonism of the glutamate receptor GluK2 suppresses dermal mast cell activation and cutaneous inflammation. Sci Transl Med.

[9] Saunders B, Elliott-Sale K, Artioli GG (2017). β-alanine supplementation to improve exercise capacity and performance: a systematic review and meta-analysis. Br J Sports Med.

